# Dual role of MdSND1 in the biosynthesis of lignin and in signal transduction in response to salt and osmotic stress in apple

**DOI:** 10.1038/s41438-020-00433-7

**Published:** 2020-12-01

**Authors:** Keqin Chen, Yunna Guo, Mengru Song, Lifu Liu, Hao Xue, Hongyan Dai, Zhihong Zhang

**Affiliations:** 1 Group of Molecular Biology of Fruit Trees, College of Horticulture, Shenyang Agricultural University, 120 Dongling Road, Shenyang, Liaoning 110866 China; 2 Group of Fruit Germplasm Evaluation & Utilization, College of Horticulture, Shenyang Agricultural University, 120 Dongling Road, Shenyang, Liaoning 110866 China

**Keywords:** Transcriptional regulatory elements, Functional genomics

## Abstract

Clarifying the stress signal transduction pathway would be helpful for understanding the abiotic stress resistance mechanism in apple (*Malus* × *domestica* Borkh.) and could assist in the development of new varieties with high stress tolerance by genetic engineering. The key NAC transcription factor SND1, which is involved in the lignin biosynthesis process in apple, was functionally analyzed. The results of the stress treatments indicated that *MdSND1* could be induced by salt, mannitol and ABA. Compared with wild-type GL-3 plants, *MdSND1*-overexpressing apple plants with greater antioxidant capacity and lignin were more resistant to salt and simulated osmotic stress, while RNAi plants were more vulnerable. Additionally, molecular experiments confirmed that MdSND1 could regulate the biosynthesis of lignin by activating the transcription of MdMYB46/83. Moreover, genes known to be involved in the stress signal transduction pathway (*MdAREB1A*, *MdAREB1B*, *MdDREB2A*, *MdRD29A*, and *MdRD22*) were screened for their close correlations with the expression of *MdSND1* and the response to salt and osmotic stress. Multiple verification tests further demonstrated that MdSND1 could directly bind to these gene promoters and activate their transcription. The above results revealed that MdSND1 is directly involved in the regulation of lignin biosynthesis and the signal transduction pathway involved in the response to both salt and osmotic stress in apple.

## Introduction

Plants live in constantly changing environments, and stressful conditions can limit their growth, development, and propagation^1^ or even cause adaptive changes in their morphology and biological processes. Generally, stressful conditions induce plant responses not only at the physiological and biochemical levels but also at the cellular and molecular levels^2^. During the molecular process underlying stress signal perception to stress-responsive gene expression, many transcription factors are involved and play roles in signal transduction^3^. Understanding and clarifying stress signaling and responses would be helpful for explaining the stress resistance mechanism in plants and would increase our ability to enhance the stress tolerance of crops to achieve agricultural sustainability and food security for an increasing global population^1^.

The NAC (NAM, no apical meristem; ATAF, *Arabidopsis* transcriptional activator and CUC, cup-shaped cotyledon) proteins, which have a highly conserved DNA-binding domain in their N-terminal region and variable domains in their C-terminal region, usually function as transcription factors in plants and play important roles in plant growth and development^4–11^. Secondary wall-associated NAC domain protein 1 (SND1), a transcription factor that functions upstream of MYB46, SND3, MYB103, and KNAT7^12^, is expressed specifically in interfibers and lignocellulosic fibers in plant stems and plays an important role in fiber thickening^13^. Simultaneous knockout of SND1/NST3 and NST1 resulted in severely reduced expression of secondary wall biosynthesis genes in conjunction with loss of all three major secondary wall components: cellulose, xylan and lignin^10,14^. In addition, NAC transcription factors have also been reported to be involved in the regulation of signal transduction and plant responses to various biotic and abiotic stresses^15–18^.

Drought and high salinity are common abiotic stresses that have an adverse effects on plant growth and productivity and can lead to deterioration of fruit quality^19,20^. Early perception of the transduction of abiotic stress signals in plants is primarily controlled by several transcription factors^21,22^ that can directly or indirectly regulate plant responses^20,23–25^. Dehydration response element-binding protein 2 (DREB2) plays an important role in the response to abiotic stress, and many DREB2 homologs have been reported to be induced by salt or drought stress, including AtDREB2A, AtDREB2B, and AtDREB2C in *Arabidopsis*;^26,27^ GmDREB2 in *Glycine max*;^28^ PeDREB2 in *Populus euphratica*;^29^ OsDREB2A in *Oryza sativa*;^30^ EsDREB2B in *Eremosparton songoricum*;^31^ and SlDREB2 in *Solanum lycopersicum*^32^. Abscisic acid-responsive element-binding protein 1 (AREB1) (also named ABF2) belongs to the basic leucine zipper (bZIP) transcription factor family and can regulate the expression of ABA-inducible genes by binding to the ABA-responsive element (ABRE) motif in their promoter. The expression of *AREB1* is upregulated not only by ABA but also in response to drought and high-salinity stress^33,34^. AREB1/ABF2 has been reported to function predominantly in regulating the expression of genes whose products function downstream of SnRK2 kinases in the ABA signaling pathway in response to osmotic stress^33^. Several stress-responsive genes, such as *RD22* and *RD29*, are induced in response to osmotic stress through AREB transcription factors in an ABA-dependent manner^34,35^.

Apple plants are widely distributed worldwide, and apple fruits have high nutritional value. Both drought and salt stress are two environmental factors limiting apple growth in some areas. Previous studies have found that the lignin biosynthesis pathway in apple is closely related to the signaling pathways of stress responses. MdMYB46, a key regulator of secondary cell wall formation, can enhance the stress tolerance of apple by directly activating stress-responsive signals^36^. Whether other types of transcription factors involved in lignin metabolism (such as NACs) have similar functions has become an interesting question for future studies. In this study, we found that, in addition to regulating the accumulation of lignin in apple plants, MdSND1 also participates in the regulation of the stress signal transduction pathway by activating the expression of stress-responsive genes.

## Results

### MdSND1 has conserved NAC DNA-binding regions

**Fig. 1:**
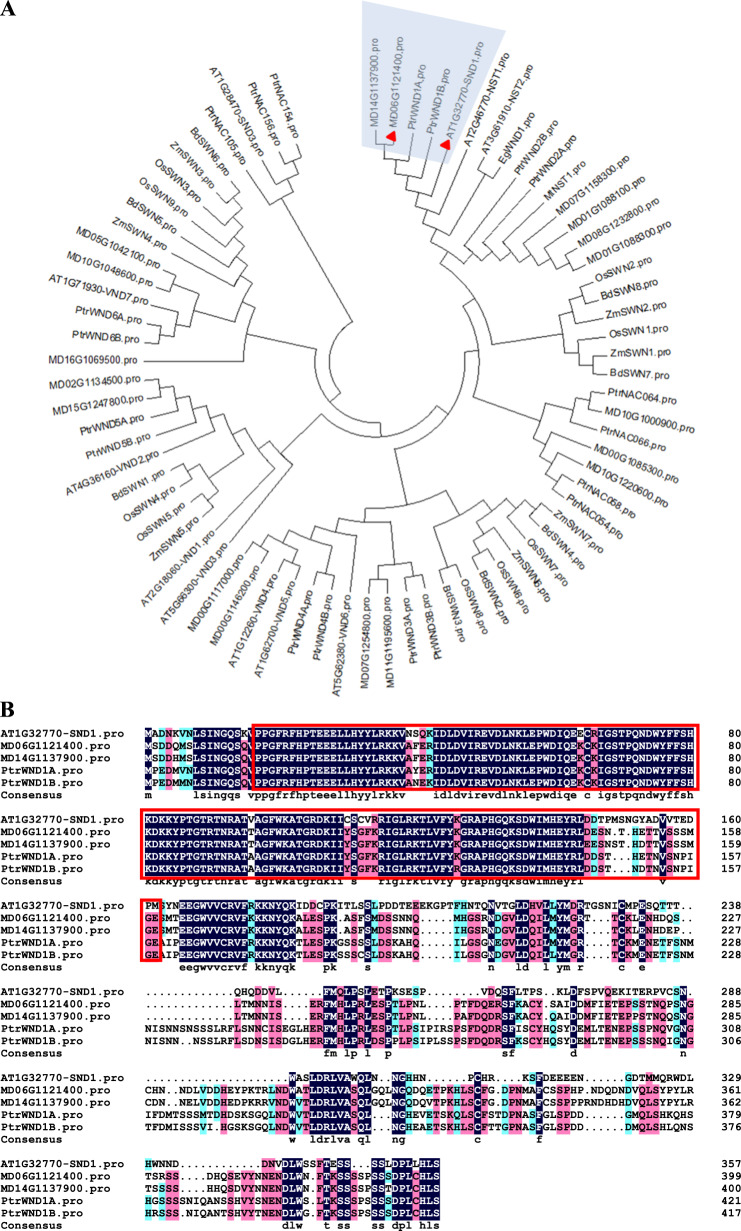
Phylogenetic analysis and sequence alignment of MdSND1. **A** Phylogenetic analysis of AtSND1 orthologs. AT (*Arabidopsis thaliana*); Mt (*Medicago truncatula*); Eg (*Eucalyptus grandis*); Os (*Oryza sativa*); Bd (*Brachypodium distachyon*); Zm (*Zea mays*); Ptr (*Populus trichocarpa*); MD (*Malus domestica*). **B** Multiple sequence alignment of AtSND1, MdSND1, and PtrWND1A/1B. The NAC DNA-binding functional regions are marked in the red box

Overexpression of *AtSND1* can increase secondary cell wall thickening in *Arabidopsis*^9^. To identify SND1 in apple, we first used the AtSND1 amino acid sequence to search for orthologs in the apple genome (GDDH13 V1.1 database). Phylogenetic analysis of apple SND1 candidate proteins (EgWND1, MtNST1, OsSWNs, ZmSWNs, BdSWNs, PtrNACs, and PtrWNDs) and *Arabidopsis* secondary cell wall NAC proteins was then carried out (Fig. 1A). The phylogenetic tree showed that both MD06G1121400 and MD14G1137900 were homologous with AtSND1, but the sequence similarity results showed that MD06G1121400 was more similar to AtSND1 (Fig. S1); therefore, MD06G1121400 was named MdSND1 in our study. The amino acid sequences of MD06G1121400, MD14G1137900, AtSND1, PtrWND1A, and PtrWND1B were also compared, and the highly conserved DNA-binding domain (indicated in the red box in the figure) was identified in all of their N-terminal sequences (Fig. 1B).

### MdSND1 functions in the nucleus and is sensitive to stress signals

**Fig. 2:**
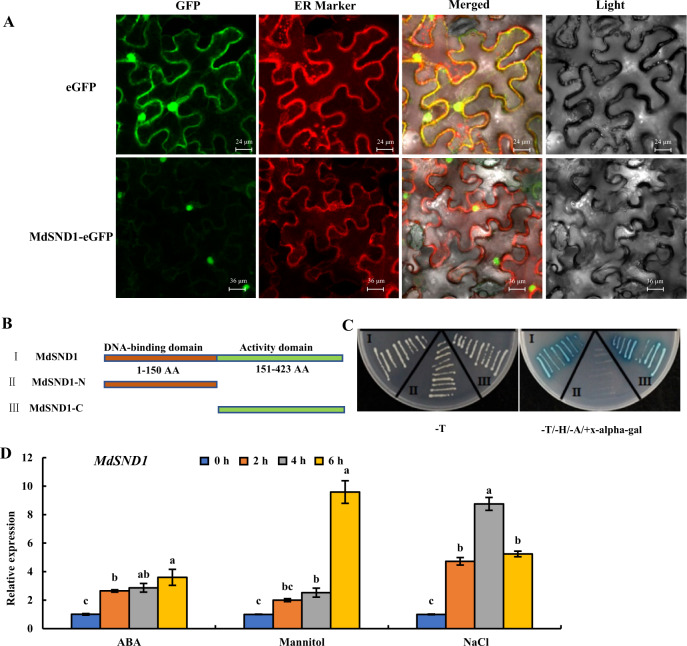
Subcellular localization, transcriptional activation, and stress induction of MdSND1 in apple. **A** Subcellular localization of pRI-MdSND1-eGFP in epidermal cells of tobacco leaves. **B** Amino acid sequence structure of full-length MdSND1, the MdSND1 N-terminal region and the MdSND1 C-terminal region. **C** Transcriptional activation assays of MdSND1. The GAL4 DNA-binding domain (GAL4DB) was fused to the MdSND1 N-terminal region, MdSND1 C-terminal region, and full-length MdSND1 sequence, after which the constructs were transferred into yeast. (−T/−H/−A+X-alpha-gal) means selective medium supplemented with 5-bromo-4-chloro-3-indolyl-alpha-D-galactoside but lacking Trp, His, and adenine, while (−T) indicates selective medium lacking Trp. **D** *MdSND1* was induced by osmotic stress. The error bars indicate the standard deviations (SDs) of three biological replicates. The letters indicate the level of significance (*P* < 0.05, according to Duncan’s multiple range test)

For subcellular localization, a pRI-MdSND1-eGFP vector was constructed on the basis of a pRI-eGFP vector and contained the *MdSND1* coding sequence region (without the stop codon). The fusion vector was then transiently introduced into *Nicotiana benthamiana* leaves via *Agrobacterium*-mediated infection. As shown in Fig. 2A, the GFP fluorescence signal revealed that MdSND1 was localized to the nucleus. Moreover, the transcriptional activation region of MdSND1 was found to be present in the C-terminal (Fig. 2B, C) by the use of a yeast two-hybrid system.

Compared with wild-type seedlings, AtSND1 deletion mutant seedlings with reduced anthocyanin contents are more sensitive to salt stress^13^. To determine the sensitivity of MdSND1 to abiotic stress, GL-3 apple plants were treated with exogenous ABA, NaCl, and mannitol. It was found that the expression levels of *MdSND1* were significantly upregulated under these stresses (Fig. 2D).

### MdSND1 positively regulates salt and simulated drought stress tolerance in apple

*MdSND1* overexpression and RNAi vectors with the CaMV 35S fragment in the promoter were transformed into GL-3 plantlets (Fig. S2A), and three *MdSND1*-overexpressing transgenic lines, namely, OE-MdSND1-5, OE-MdSND1-7, and OE-MdSND1-16, and five MdSND1-RNAi lines of GL-3 apple, namely, RNAi-MdSND1-1, RNAi-MdSND1-3, RNAi-MdSND1-10, RNAi-MdSND1-12, and RNAi-MdSND1-17, were obtained. The transcript levels of *MdSND1* in the three overexpressing apple lines significantly increased (Fig. S2B), while those in the *MdSND1-*RNAi lines significantly decreased (Fig. S2C).

**Fig. 3:**
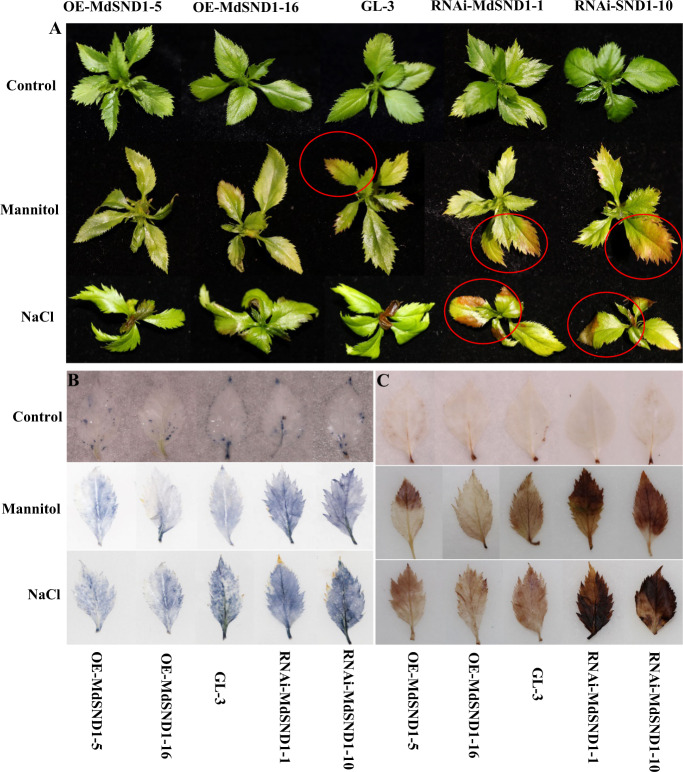
MdSND1 enhanced the tolerance of transgenic apple plants to salt and osmotic stress. **A** *MdSND1*-overexpressing and *MdSND1*-RNAi apple plants showed different phenotypes under salt and osmotic stresses. Two hundred millimolar NaCl or 300 mM mannitol was added to the medium when the nontransgenic GL-3 apple plants and transgenic apple were twenty days old. **B** Detection of ROS levels in apple leaves of plants under salt and mannitol treatment for 10 days according to nitro blue tetrazolium (NBT) staining. The darker the blue precipitate is, the higher the ROS level. **C** Detection of ROS levels in apple leaves of plants under salt and mannitol treatment for 10 days according to 3,3’-diaminobenzidine (DAB) staining. The darker the brown precipitate is, the higher the ROS level

The OE-MdSND1-5, OE-MdSND1-16, RNAi-MdSND1-1, and RNAi-MdSND1-10 transgenic apple lines and wild-type apple plants (GL-3) were subjected to long-term stress treatments to explore the function of MdSND1 in apple. The apple plants showed different phenotypes in response to abiotic stress. Under the salt treatment, the leaves of the *MdSND1*-RNAi apple plants were yellowish-brown and severely curled, while those of the *MdSND1*-overexpressing plants were still green, comparable to those of the wild-type plants (red circle in Fig. 3A). Similarly, there were no obvious phenotypic changes in the leaves of the *MdSND1*-overexpressing plants under mannitol stress treatment, while the leaves of the *MdSND1*-RNAi plants showed a large area of browning. Based on the above description, the *MdSND1*-overexpressing apple plants appeared to be more resistant to both salt and osmotic stress than the wild-type plants did, while the *MdSND1*-RNAi plants were more vulnerable.

Reactive oxygen species (ROS) act as important molecules during plant stress responses and play a key role in activating downstream metabolic pathways. Since 3,3’-diaminobenzidine (DAB) and nitro blue tetrazolium (NBT) can produce reddish-brown and blue precipitates, respectively, after reacting with ROS, they were used to detect H_2_O_2_ and superoxide (O_2_^−^) anions in the leaves of apple plants after stress treatments. The DAB and NBT staining results showed that there were lower levels of ROS in the leaves of *MdSND1*-overexpressing apple plants compared with the nontransgenic plants, while there were greater levels in the *MdSND1*-RNAi plants (Fig. 3B, C).

**Fig. 4:**
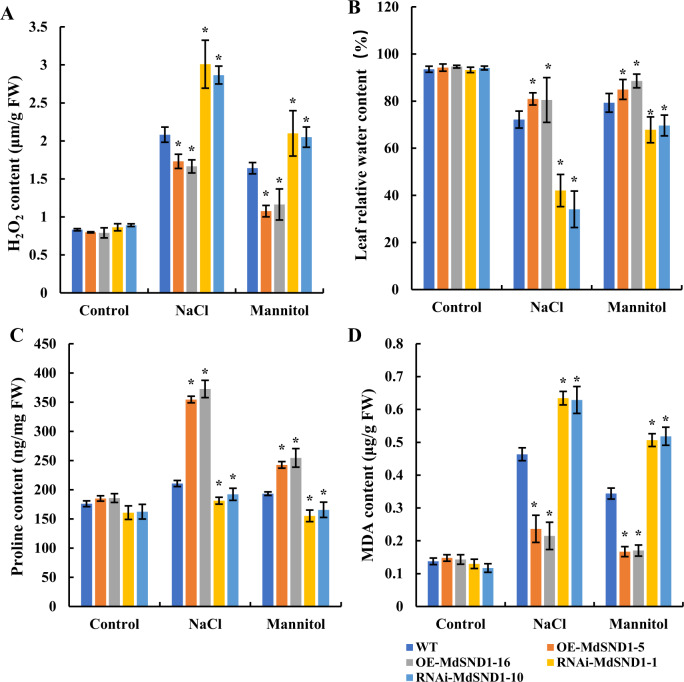
Comparison of the content of stress-related metabolites in transgenic apple plants under salt and osmotic stress. **A** Content of H_2_O_2_. **B** Relative water content. **C** Content of proline. **D** Content of malondialdehyde (MDA). The error bars indicate the standard deviations (SDs) of three biological replicates, *P < 0.05 (according to *t*-tests)

The contents of stress-related metabolites in apple plants under both salt and osmotic stress are presented in Fig. 4. Like the results of the DAB/NBT staining showed, the content of H_2_O_2_ in the leaves of the *MdSND1*-overexpressing apple plants was lower than that in the nontransgenic plants, while it was the highest in the *MdSND1*-RNAi plants (Fig. 4A). The relative water content of the leaves is a potential indicator of plant water loss under abiotic stress. The relative water content of the MdSND1-overexpressing apple plants was the highest after exposure to salt and osmotic stress (Fig. 4B). The proline content was positively correlated with stress resistance; proline plays a role in preventing cell dehydration. Compared with that in wild-type GL-3 plants, the proline content in the MdSND1-overexpressing apple plants was highest, while it was lowest in the RNAi plants (Fig. 4C). MDA is a product of plant membrane lipid peroxidation and indicates the degree of cell membrane lipid peroxidation. Under abiotic stress, there was less MDA accumulation in the *MdSND1*-overexpressing apple leaves than in the wild-type GL-3, while there was more MDA accumulation in the RNAi plants (Fig. 4D).

### The transcript level of the secondary cell wall formation key regulatory genes *MdMYB46/83* in apple are positively regulated by MdSND1

Since the lignin content showed a strong correlation with the transcript level of *MdSND1* (Fig. S3A), the effect of MdSND1 on lignin metabolism was investigated. The expression levels of lignin biosynthesis-related MYB transcription factors and structural genes in transgenic apple plants were examined. The transcript levels of lignin biosynthesis genes (*MdCCR*, *MdCOMT*, *MdHCT*, *MdF5H*, *Md4CL*, *MdCAD*, *MdC3H*, and *MdC4H*) and MYB genes (*MdMYB46*, *MdMYB83A*, and *MdMYB83B*) were significantly upregulated in the *MdSND1*-overexpressing lines but were downregulated in the RNAi lines (Fig. S3B, C).

Previous studies have shown that MYB83 and MYB46 redundantly regulate secondary wall formation in fibers and vessels and are direct targets of SND1^37^. To clarify the regulatory role of MdSND1 in the formation of secondary cell walls, we analyzed the promoter region of the *MdMYB46* and *MdMYB83* genes.

**Fig. 5:**
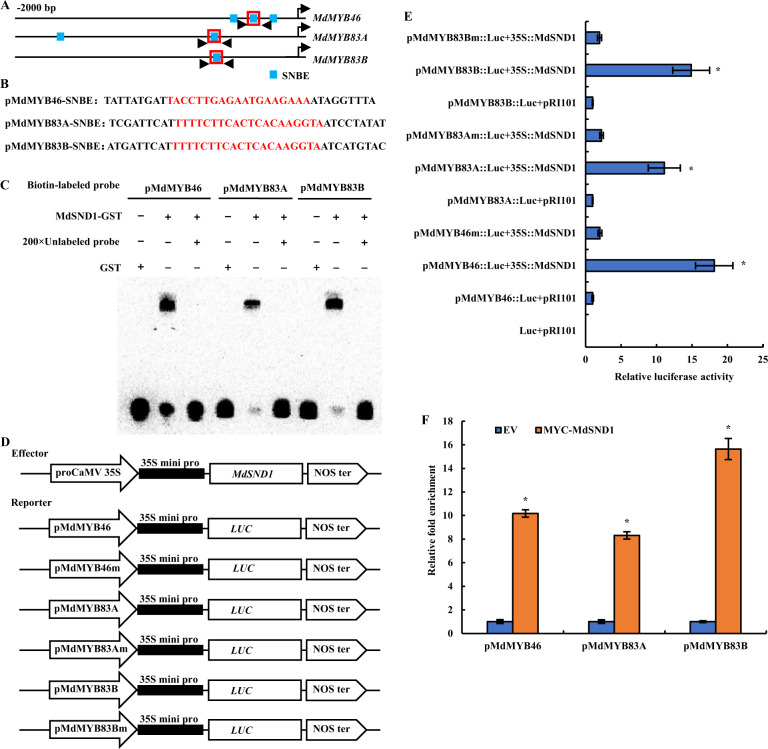
Binding of MdSND1 to SNBE motifs in the promoters of the *MdMYB46* and *MdMYB83* genes. **A** MdSND1-binding sites in the *MdMYB46 and MdMYB83* gene promoters. **B** SNBE site sequences in the *MdMYB46*, *MdMYB83A*, and *MdMYB83B* promoters. The SNBE sequence is marked in red. **C** EMSA showing the competitive binding of MdSND1 to SNBE fragments with unlabeled DNA probe (200-fold). **D** Vector structure of the effector and reporter used for tobacco transient expression assays. **E** Relative luciferase activity in the transient expression assays. **F** ChIP-qPCR-based analysis showing MdSND1 binding to the promoters of *MdMYB46* and *MdMYB83*. The error bars indicate the standard deviations (SDs) of three biological replicates, *P < 0.05 (according to *t*-tests)

SND1 can bind specifically to secondary wall NAC-binding element (SNBE) sites in its downstream gene promoter^9,10^. Multiple SND1-binding sites were found in the promoters of the lignin biosynthesis regulatory genes (*MdMYB46*, *MdMYB83A*, and *MdMYB83B*) (Fig. 5A). To investigate whether MdSND1 binds to the MdMYB46 and MdMYB83 promoters, we applied EMSAs that involved a GST-SND1 fusion protein and an SNBE promoter fragment (Fig. 5B). It was found that the recombinant MdSND1 protein was able to bind to the *MdMYB46* and *MdMYB83* promoter fragments, causing a mobility shift (Fig. 5C). However, mobility shifts were not seen when the 200-fold unlabeled *MdMYB46/MdMYB83* promoter fragments competed with the biotin-labeled promoter fragments and when the *MdMYB46/MdMYB83* promoter fragment was incubated with GST alone (Fig. 5C), indicating that the binding of MdSND1 to the *MdMYB46* and *MdMYB83* promoters was specific.

In addition, fragments (approximately 200–500 bp) of the promoters of the *MdMYB46*, *MdMYB83A*, and *MdMYB83B* genes containing SNBE sites and the mutated SNBE sites (shown by the black arrow in Fig. 5D) were integrated into a luciferase reporter vector. p35S::MdSND1 was then injected together with these reporters as an effector, and the luciferase activity in tobacco leaves was evaluated after 48 h. The above results indicated that MdSND1 could activate transcription of the *MdMYB46* and *MdMYB83* genes (Fig. 5E).

To further verify the above results, a chromatin immunoprecipitation test was used to examine whether MdSND1 could bind to the promoters of MdMYB46 and MdMYB83 in vivo. We first overexpressed MYC-tagged SND1 in Ourin apple calluses. An anti-MYC antibody was then used to immunoprecipitate chromatin from MYC-SND1 overexpressors cross-linked by formaldehyde for enrichment of MYC-SND1-bound DNA fragments. Finally, we used the immunoprecipitated DNA fragments as templates for qRT-PCR-based detection of *MdMYB83* and *MdMYB46* promoter sequences. The results showed that the fragments in the promoters of *MdMYB46*, *MdMYB83A*, and *MdMYB83B* contained SNBE sites, which could be strongly bound by MdSND1 (Fig. 5F).

From the in vitro and in vivo binding analyses, we can conclude that MdSND1 directly binds to the promoters of the *MYB46* and *MYB83* genes to regulate their expression. In addition, we also analyzed the promoters of several lignin biosynthetic genes (*MdCCR*, *MdCOMT*, *MdHCT*, *MdF5H*, *Md4CL*, *MdCAD*, *MdC3H*, and *MdC4H*) (Fig. S4) and found that there were multiple SNBE sites in these genes (except *MdC4H*), which indicated that these genes might also be directly regulated by MdSND1.

### Expression of some stress-responsive genes is induced by MdSND1 in apple

As shown in Fig. 2D, the expression of *MdSND1* can be induced by ABA, NaCl, and mannitol. To determine the function of MdSND1 in the stress response, the relationships between MdSND1 and our prescreened stress response genes (*MdAREB1A*, *MdAREB1B*, *MdDREB2A*, *MdRD22*, *MdRD29A*, and *MdRD29B*)^36^ were investigated.

**Fig. 6:**
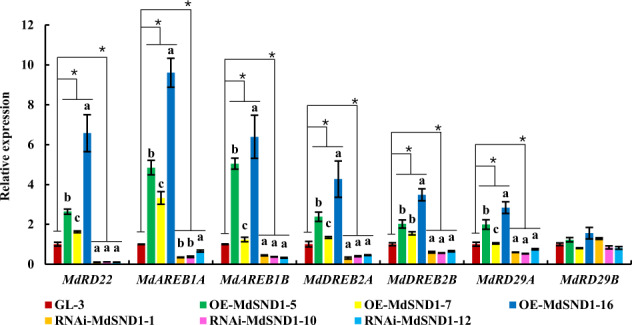
Transcript levels of genes related to stress signaling in MdSND1 transgenic apple. The error bars indicate the standard deviations (SDs) of three biological replicates, *P < 0.05 (according to *t*-tests). The letters indicate the level of significance (*P* < 0.05, according to Duncan’s multiple range test)

The transcript levels of these stress-responsive genes in *MdSND1* transgenic and wild-type apple plants were measured. As shown in Fig. 6, the transcript levels of the *MdAREB1A*, *MdAREB1B*, *MdDREB2A*, *MdRD22*, and *MdRD29A* genes were positively correlated with the level of *SND1* in apple plants, but the transcript level of *MdRD29B* changed statistically insignificantly in the overexpression and RNAi plants, indicating that the transcription of the *MdAREB1A*, *MdAREB1B*, *MdDREB2A*, *MdRD22*, and *MdRD29A* genes could be affected by MdSND1 in apple.

### MdSND1 activates the transcription of stress signaling genes by directly binding to SNBE motifs in their promoters

**Fig. 7:**
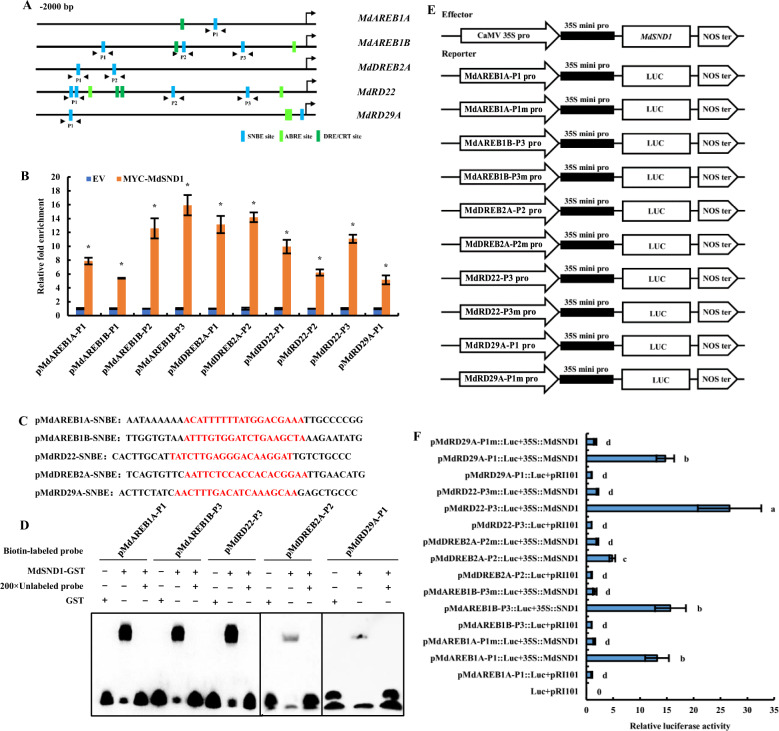
MdSND1 can directly bind to the SNBE motif in the promoter of stress signaling genes. **A** Positions of SNBEs, DRE/CRTs, and ABREs in the promoters of *MdAREB1A*, *MdAREB1B*, *MdDREB2A*, *MdRD22*, and *MdRD29A*. **B** ChIP-qPCR assay showing MdSND1 binding to the promoters of *MdAREB1A*, *MdAREB1B*, *MdDREB2A*, *MdRD22*, and *MdRD29A*. The error bars indicate the standard deviations (SDs) of three biological replicates, *P < 0.05 (according to *t*-tests). **C** SNBE sequence in the promoter of stress-responsive signaling genes. The SNBE sequence is marked in red. **D** EMSA results showing the competing binding of MdSND1 to SNBE fragments with unlabeled DNA probe (200-fold). **E** Vector structure of the effector and reporter used for the tobacco transient expression assays. **F** Relative luciferase activity in the transient expression assays. The error bars indicate the standard deviations (SDs) of three biological replicates. The letters indicate the significance level (*P* < 0.05, according to Duncan’s multiple range test)

The sequences of 2000 bp of the promoters of *MdRD29A*, *MdAREB1B*, *MdRD22*, *MdAREB1A*, and *MdDREB2A* were downloaded (https://iris.angers.inra.fr/gddh13/jbrowse/?data=gddh13) and analyzed, the results of which are shown in Fig. 7A. There were four SNBE sites in the promoter of *MdRD22*, two SNBE sites in the promoter of *MdRD29A*, one SNBE site in the promoter of *MdAREB1*, three SNBE sites in the promoter of *MdAREB1B*, and two SNBE sites in the promoter of *MdDREB2A*, indicating that they all had the potential to be regulated by MdSND1.

To verify the interactions between MdSND1 and the SNBE binding sites in the promoters of *MdAREB1A*, *MdAREB1B*, *MdDREB2A*, *MdRD22*, and *MdRD29A*, a ChIP approach was used to examine whether MdSND1 binds to these promoters in vivo. MYC-tagged *SND1* was overexpressed in Ourin apple calli. After the steps of formaldehyde cross-linking chromatin and MYC antibody precipitating DNA fragments, we used the immunoprecipitated DNA fragments as templates for qRT-PCR for detection of *MdRD29A*, *MdAREB1A*, *MdAREB1B*, *MdRD22*, and *MdDREB2A* promoter sequences. MdSND1 was found to bind to these sites to varying degrees (Fig. 7B).

The SNBE sequence (Fig. 7C) with the strongest binding ability among the MdAREB1A-P1, MdAREB1B-P3, MdDREB2A-P2, MdDR22-P3, and MdRD29A-P1 fragments was selected for designing biotin-labeled probes based on the results of the ChIP-qPCR. These probes were then incubated with GST-MdSND1 proteins. We used an electrophoretic mobility shift assay to detect binding between MdSND1 and the promoter fragments and an unlabeled probe (200-fold) for a competition assay. Fig. 7D shows that the promoters of the *MdAREB1A*, *MdAREB1B*, *MdDREB2A*, *MdRD22*, and *MdRD29A* genes were bound to the MdSND1 protein.

We also inserted the fragments from the promoters of *MdAREB1A-P1*, *MdAREB1B-P3*, *MdDREB2A-P2*, *MdDR22-P3*, and *MdRD29A-P1* and the corresponding mutated SNBE binding site sequences into a luciferase-containing reporter gene vector for further verification tests (Fig. 7E). The reporter vector was inserted into the leaves of tobacco together with either the effector p35S::MdSND1 or a pRI101AN empty vector. The fluorescence distribution and luciferase activity results showed that MdSND1 could directly activate the expression of the *MdAREB1A*, *MdAREB1B*, *MdDREB2A*, *MdRD22*, and *MdRD29A* genes by binding to their promoters.

## Discussion

In *Arabidopsis* woody tissue, the key regulators of secondary wall formation are NST1 and SND1 (also known as NST3). SND1 and NST1 are functionally redundant in regulating secondary wall biosynthesis^10,14^, and the secondary wall thickening of interfibers and secondary xylem is completely inhibited in mutants with mutations in both of these genes^10^. AtSND1 has a dual role in plant growth: it either directly binds to the MYB46 promoter region, thus activating the lignin biosynthesis pathway, or directly binds to the ABI4 gene promoter, thereby maintaining low levels of ABA and inhibiting growth^13^. In the present study, MdSND1 and AtSND1 showed some functional similarities in regulating lignin synthesis. The lignin content increased in apple plants overexpressing MdSND1 but decreased in RNAi plants (Fig. S3). MdSND1 was able to activate the transcription of *MdMYB46/83* and indirectly regulate the accumulation of lignin in apple. However, whether SND1 can function like MdMYB46 and directly act on the promoter of lignin biosynthesis genes remains unknown^36^.

**Fig. 8:**
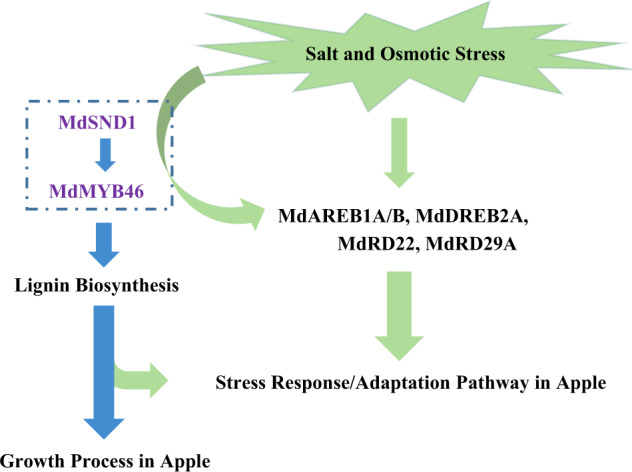
Functional model of MdSND1 in apple. MdSND1 not only regulates lignin biosynthesis but also enhances both salt and osmotic stress tolerance by directly activating stress-responsive signals. The blue arrow represents the regulation of the lignin biosynthesis process, and the green arrow represents the regulation of the stress response process

Both drought and salt stress can lead to changes in osmotic pressure in cells, while salt also has additional ionic or ion-toxicity effects^1,38^. Elucidating the signal transduction response pathway involved in both salt and osmotic stress is of great significance for clarifying the antistress mechanism of plants. This knowledge is essential for guiding the development of varieties with broad adaptability. In the present study, the transcription of *MdSND1* was induced by abiotic stress (Fig. 2D). Moreover, the *MdSND1*-overexpressing plants showed higher salt and osmotic stress tolerance than did the nontransgenic plants, while the *MdSND1*-RNAi plants were more vulnerable, similar to the AtSND1 deletion mutant seedlings, indicating that the transcript level of *MdSND1* is intrinsically linked to the stress tolerance of apple. A series of stress-responsive pathways in plants is triggered under salt and osmotic stress^1,20^, in which both ABA-dependent and ABA-independent regulatory systems are involved^2^. The bZIP TF AREB and the DREB AP2/ERF TF family members have pivotal functions in ABA-dependent and ABA-independent gene expression, respectively^39–42^. In addition, several stress-responsive genes, such as *RD22* and *RD29A*, are also activated by dehydration, high salinity, and cold stress and function in signal transduction pathways^38,43^. In apple, *MdRD29A*, *MdRD29B*, *MdAREB3.2* (*MdAREB1A*), *MdAREB2* (*MdAREB1B)*, *MdDREB2A*, *MdDREB2B*, and *MdRD22* were recently found to be responsive to stress signals^44–47^. In this study, the transcript levels of *MdRD22*, *MdAREB1A*, *MdRD29A*, *MdAREB1B*, and *MdDREB2A* were up- or downregulated in *MdSND1*-overexpressing or -RNAi apple plants, respectively (Fig. 6), and ChIP assays, EMSAs and luciferase reporter transcription activity assays further confirmed that MdSND1 could bind to the SNBE motifs in the promoters of the five genes and activate their expression (Fig. 7), which indicates that MdSND1 could have a role in activation of stress signal transduction pathways (Fig. 8).

SND1 belongs to the NAC superfamily, which is one of the largest plant-specific transcription factor families^48^. NAC TFs regulate the transcription of downstream target genes by binding to multiple motifs, such as NAC-binding site (*NACBS*s), in which GCTT is the core binding motif^49,50^, the core sequence CA(A/C)G(T/C) (T/C/A) (T/C/A)^51^ and SNBEs, in which (T/A)NN(C/T) (T/C/G)TNNNNNNNA(A/C)GN(A/C/T)(A/T) is the consensus sequence^52^. Several NAC transcription factors in different plant species have been shown to be closely correlated with the stress-related signal transduction pathway and with abiotic stress tolerance^15,18,48^. In *Arabidopsis thaliana*, by binding to the TTGCGTA motif in its promoter, ATAF1 regulates the transcription of NCED3, which encodes the key enzyme in ABA biosynthesis under dehydration stress^53,54^. AtANAC016 has been reported to be a positive regulator of plant tolerance to drought stress^55^. In apple, salt stress induces the expression of MdNAC047, which promotes the accumulation of ethylene by activating the expression of MdERF3, modulating plant salt tolerance^45^. In our study, MdSND1 could activate the transcription of stress-responsive genes by directly binding to SNBE sites, which reaffirms the important role of NAC regulators in regulating the stress signal transduction pathway.

## Conclusions

In this study, the key NAC transcription factor SND1, which is involved in the lignin biosynthesis process, was functionally analyzed in apple. MdSND1 could promote the accumulation of lignin and enhanced both salt and osmotic stress tolerance in apple. MdSND1 regulates the biosynthesis of lignin by activating the transcription of *MdMYB46/83* and participates in the stress signal transduction pathway by directly activating stress-response signals (*MdAREB1A*, *MdAREB1B*, *MdDREB2A*, *MdRD29A*, and *MdRD22*).

## Methods

### Plant materials

Tissue culture-generated GL-3 apple plants were selected from *Malus* × *domestica* cv. Royal Gala seedlings in our laboratory^56^. GL-3 plantlets were subcultured in medium (MS medium supplemented with 0.3 mg/L 6-BA, 0.2 mg/L IAA and 0.1 mg/L GA_3_) under long-day conditions (14 h light:10 h dark) at 25 °C.

### Vectors and transformation

The DNA sequence of *MdSND1* in GL-3 was the same as that of MD06G1121400 in the apple genome (https://iris.angers.inra.fr/gddh13/jbrowse/?data=gddh13). *MdSND1* overexpression and RNAi vectors were constructed for apple transformation according to the methods of Chen et al.^36^. The overexpression and RNAi vectors of *MdSND1* were then introduced into *Agrobacterium tumefaciens* strain EHA105 for GL-3 transformation^56^. The primers used are listed in Table S1.

The pRI-MYC-MdSND1 vector for callus transformation was constructed on the basis of the pRI-MdSND1 vector, with the MYC tag added before the *MdSND1* full-length CDS. The fusion vector was then introduced into EHA105 for Ourin callus transformation^56^, after which a successfully transformed callus was used for subsequent chromatin immunoprecipitation.

### Stress treatments; measurements of lignin, proline, H_2_O_2_, MDA and relative water content; and NBT/DAB staining

The following were performed as described in ref.^36^: short/long-term stress treatments; lignin, proline, H_2_O_2_, MDA and relative water content measurements; and NBT/DAB staining.

### ChIP assays

A 20-day-old positive callus transformed with pRI-MYC-MdSND1 was screened five times before being used for chromatin immunoprecipitation experiments via a ChIP-qPCR kit (Farmingdale, USA) according to the manufacturer’s instructions^36^. For ChIP-quantitative PCR analysis, *MdACTIN* was used as an endogenous control for normalization, and the fold enrichment of MdSND1-bound DNA fragments was calculated by comparing the samples with the inputs. All the primers used are listed in Table S1.

### EMSAs

The full-length *MdSND1* CDS region fused to a GST tag was transformed into *Escherichia coli* BL21 to produce a GST-MdSND1 recombinant protein, which was further purified and incubated together with SNBE oligonucleotides marked with biotin-11-UTP-labeled DNA fragments from the promoters of osmotic stress-responsive genes (*MdAREB1A*, *MdAREB1B*, *MdDREB2A*, *MdRD22*, and *MdRD29A*) for 30 min. The DNA signals were detected by chemiluminescence (Biyuntian, Shanghai, China). For the competition assays, unlabeled oligonucleotides (labeled probes at 200-fold) were added to the above binding buffer (Biyuntian).

### Analysis of transcription activity

The 200–500 bp fragments containing the corresponding binding sites in the promoters of *MdAREB1A*, *MdAREB1B*, *MdDREB2A*, *MdRD22*, and *MdRD29A* were cloned and inserted into a pRI-mini35S-LUC vector. The newly constructed pRI-mini35S-LUC vector was used as a reporter; the pRI-MdSND1 vector served as an effector; and pRI-mini35S-LUC vectors containing mutated conserved binding sites from the *MdAREB1A*, *MdAREB1B*, *MdDREB2A*, *MdRD22*, and *MdRD29A* promoter fragments were used as controls. The transcription activation assay was performed according to a previously described method^36^.

### RNA extraction and qRT-PCR

The methods of RNA extraction and qRT-PCR were the same as previously described ones^36^.

## Supplementary information

Supplemental Materials

## Data Availability

The datasets used and/or analyzed during the current study are available from the corresponding author upon reasonable request. The genes sequences can downloaded from the NCBI database, and the genes and their accession numbers are as follows: *MdMYB46* (XM_008365407.3), *MdMYB83B* (XM_008346410.3), *MdMYB83A* (XM_008376953.3), *MdMYB63* (XM_008384900.3), *MdMYB58* (XM_008373948.3), *MdDREB2B* (MG099825.1), *MdDREB2A* (XM_008355947.3), *MdAREB1B* (XM_008374912.3), *MdAREB1A* (XM_029094247.1), *MdRD29B* (XM_008378353.3), *MdRD29A* (XM_008345499.3), *MdRD22* (XM_017333810.2), *MdCCR* (XM_008377320.3), *MdCOMT* (XM_008387517.3), *Md4CL* (XM_029091143.1), *MdHCT* (XM_008344601.3), *MdF5H* (XM_008339691.3), *MdC3H* (XM_008380681.3), *MdC4H* (NM_001294106.1), *PtrWND1A* (HQ215847), *PtrWND1B* (HQ215848), *PtrWND2A* (HQ215849), *PtrWND2B* (HQ215850), *PtrWND3A* (HQ215851), *PtrWND3B* (HQ215852), *PtrWND4A* (HQ215853), *PtrWND4B* (HQ215854), *PtrWND5A* (HQ215855), *PtrWND5B* (HQ215856), *PtrWND6A* (HQ215857), *PtrWND6B* (HQ215858), *PtrNAC054* (XM_002316127), *PtrNAC058* (XM_002311240), *PtrNAC064* (XM_002319836), and *PtrNAC066* (XM_002329142), *PtrNAC154* (XM_002327995), *PtrNAC156* (XM_002309731), *PtrNAC105* (XM_002329769), *EgWND1* (HQ215846), *MtNST1* (GU144511), *OsSWN1* (JN634070), *OsSWN2* (JN634071), *OsSWN3* (JN634072), *OsSWN4* (JN634073), *OsSWN5* (JN634074), *OsSWN6* (JN634075), *OsSWN7* (JN634076), *OsSWN8* (XM_006647538), *OsSWN9* (XM_006658959), *BdSWN1* (JQ693422), *BdSWN2* (JQ693423), *BdSWN3* (JQ693424), *BdSWN4* (JQ693425), *BdSWN5* (JQ693426), *BdSWN6* (JQ693427), *BdSWN7* (JQ693428), *BdSWN7* (JQ693429), *ZmSWN1* (JN634077), *ZmSWN2* (JN634078), *ZmSWN3* (JN634079), *ZmSWN4* (JN634080), *ZmSWN5* (JN634081), *ZmSWN6* (JN634082), and *ZmSWN7* (JN634083).
