# Supplementary material for: Dual role of MdSND1 in the biosynthesis of lignin and in signal transduction in response to salt and osmotic stress in apple
Source: Hortic Res. 2020 Dec 1;7:204. doi: 10.1038/s41438-020-00433-7 (PMC7705020; doi:10.1038/s41438-020-00433-7)
Supplement: Supplementary file 1 — Supplemental Materials [file 41438_2020_433_MOESM1_ESM.pdf]

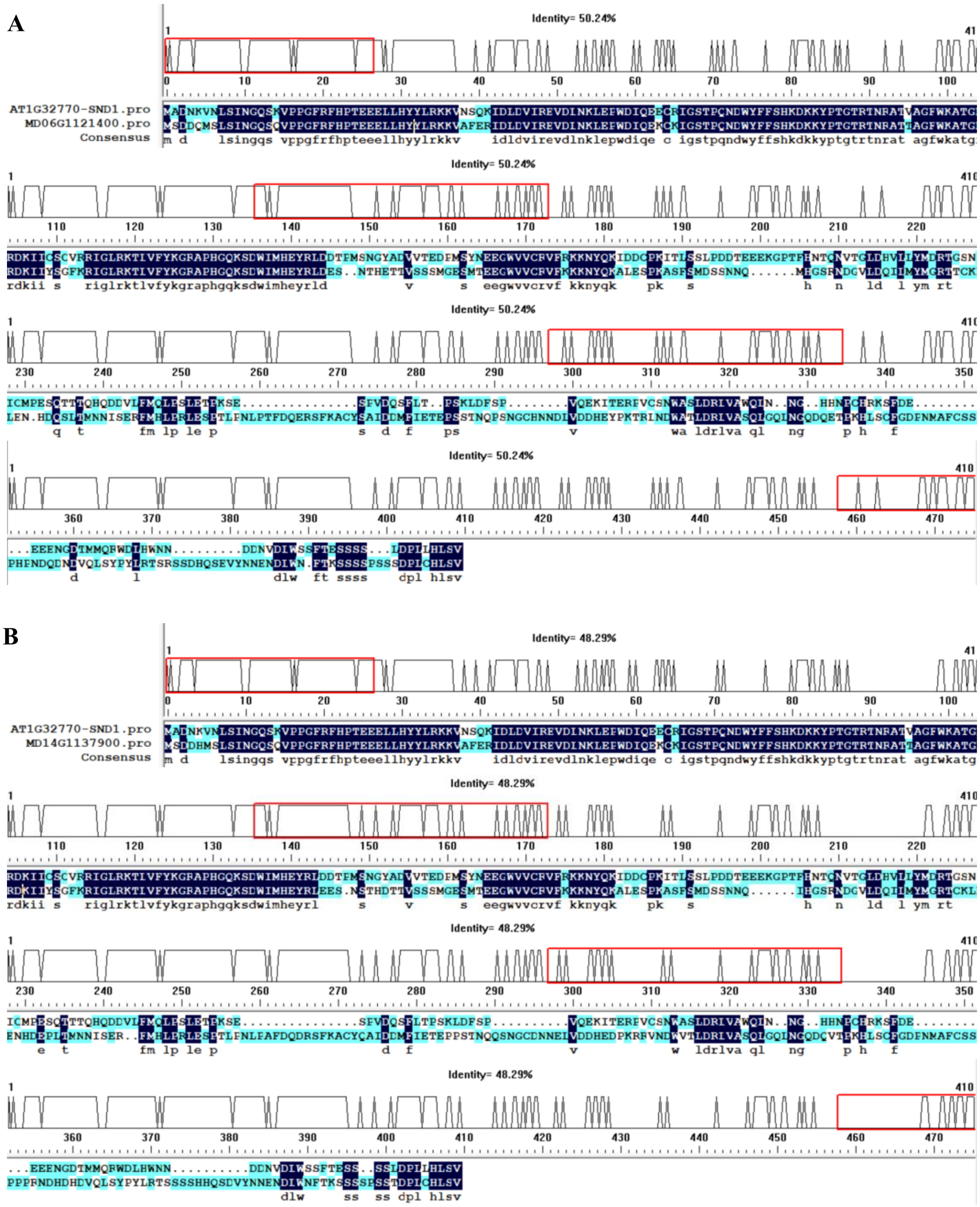

Fig. S1. Multiple sequence alignments of AtSND1 with MD06G1121400 (A) and MD14G1137900 (B).

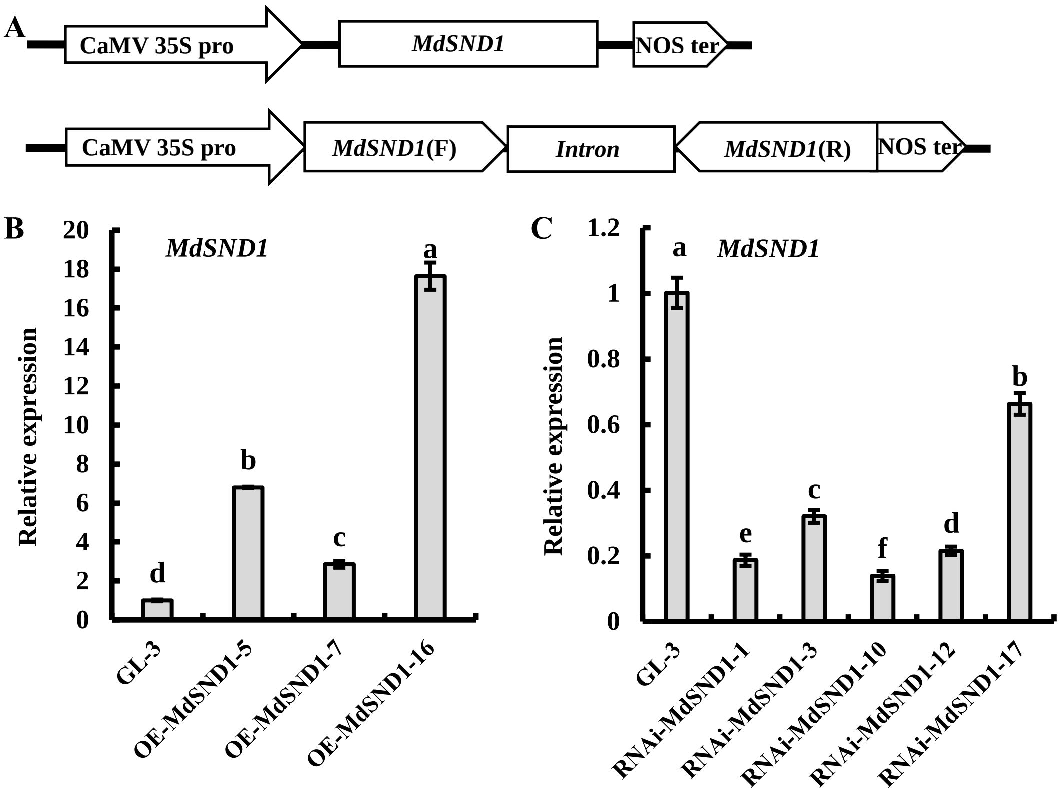

Fig. S2. Transcriptional levels of *MdSND1* in transgenic apple. A. Schematic shown the overexpressing and RNAi vector of *MdSND1*. B. *MdSND1* expression levels in non-transgenic GL-3 apple plants and *MdSND1*-overexpressing apple plants (OE-MdSND1). C. *MdSND1* expression levels in non-transgenic GL-3 apple plants and *MdSND1*-RNAi plants (RNAi-MdSND1). The error bars indicate the standard deviation (SD) from three biological replicates. The letters indicate the level of difference ($P < 0.05$, based on Duncan's multiple range test).

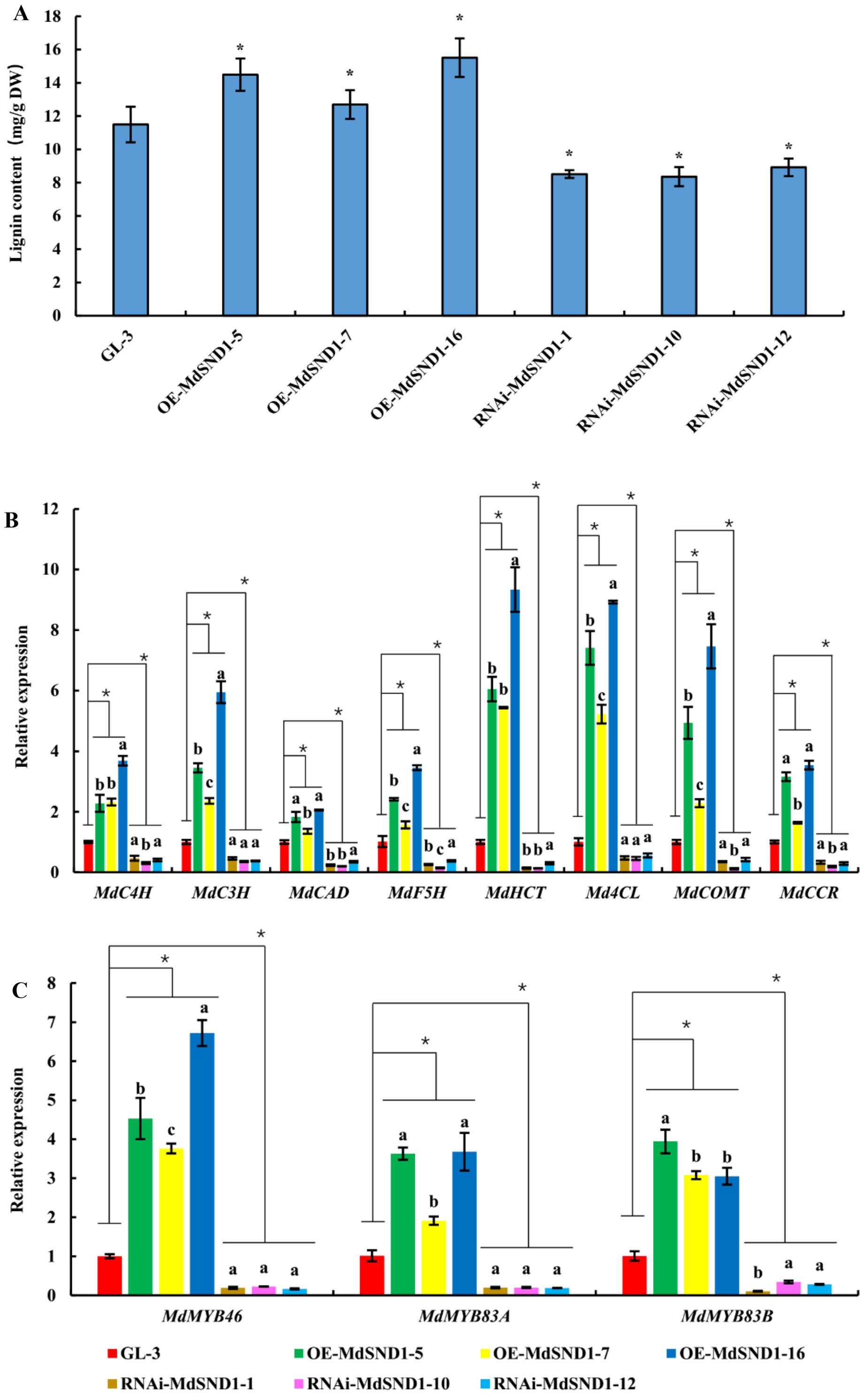

Fig. S3. The lignin content and the expression of lignin biosynthesis related genes in *MdSND1* transgenic apple. A. The content of lignin in *MdSND1* transgenic apples. B. Transcriptional levels of *MdMYB46* and *MdMYB83* in *MdSND1* transgenic apples. C. Transcriptional levels of lignin biosynthesis related genes in *MdSND1* transgenic apples. The error bars indicate the standard deviation (SD) from three biological replicates, *$P < 0.05$ (based on t-test). The letters indicate the level of difference ($P < 0.05$, based on Duncan's multiple range test).

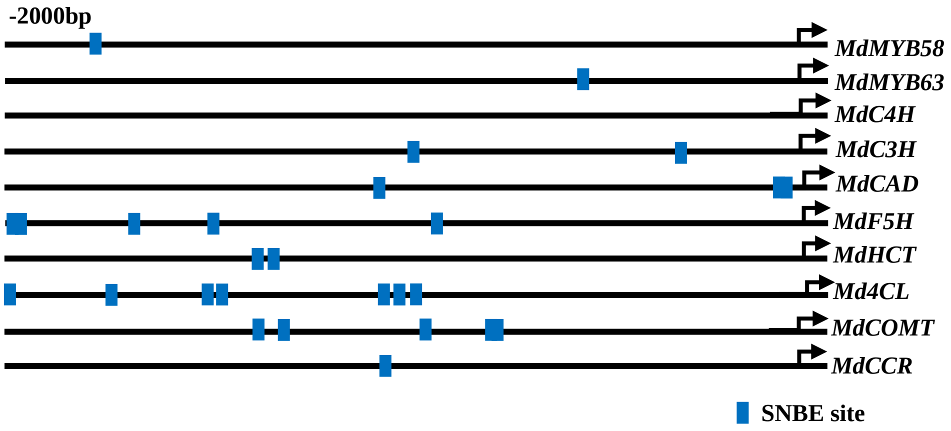

Fig. S4 The SNBE site analysis in the promoter of lignin biosynthesis genes.

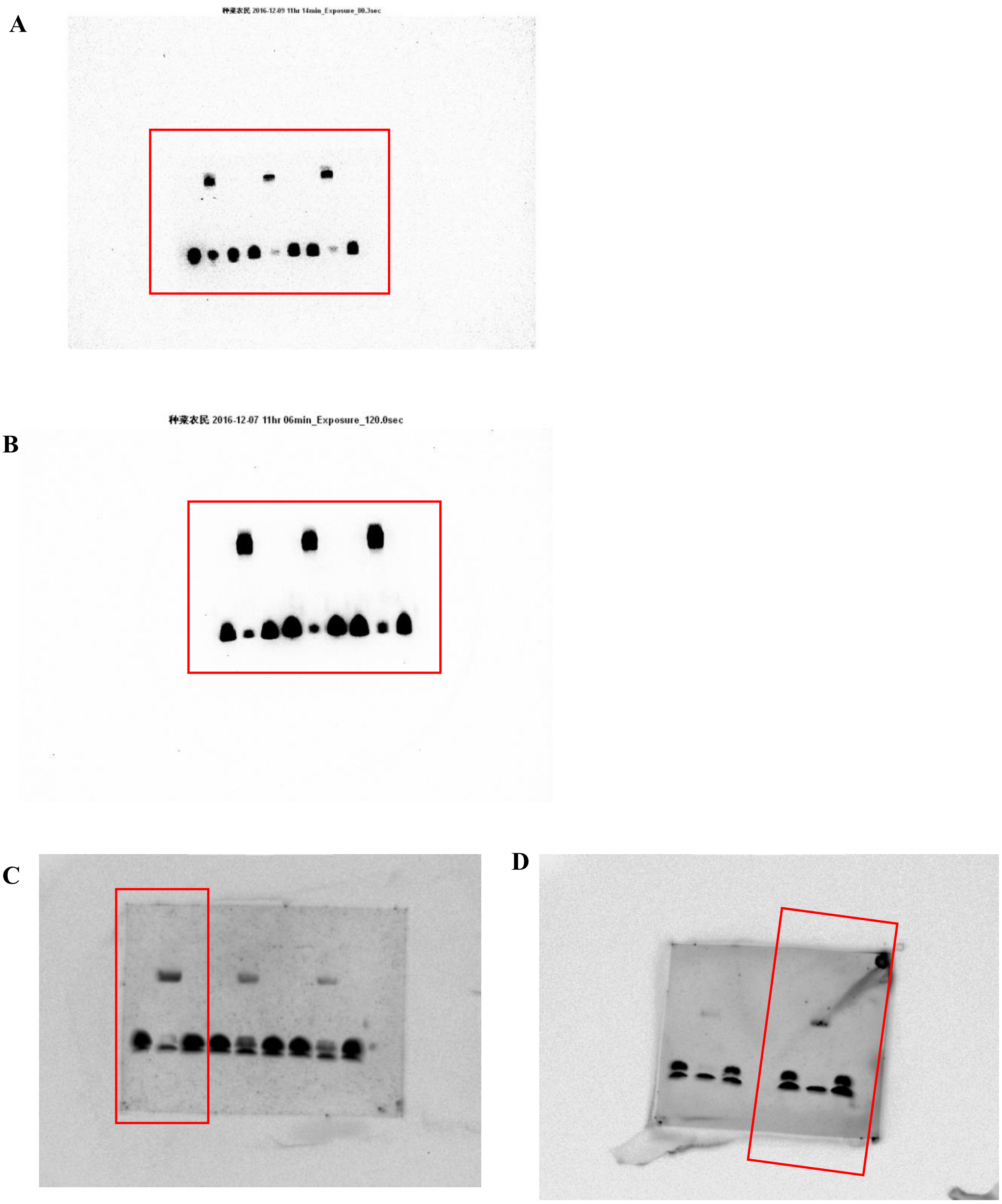

Fig. S5. Original gels images used in the study

A. The original imagine in Fig. 5C. The original gels in the red frame correspond that in Fig. 5C.

B. The original imagine of the binding blot on pMdAREB1A-P1, pMdAREB1B-P3, pMdRD22-P3 in Fig. 7C. The original gels in the red frame correspond the part of pMdAREB1A-P1, pMdAREB1B-P3, pMdRD22-P3 in Fig. 7C.

C. The original imagine of the binding blot on pMdDREB2A-P2. The original gels in the red frame correspond the part of pMdDREB2A-P2 in Fig. 7C.

D. The original imagine of the binding blot on pMdRD29A-P1. The original gels in the red frame correspond the part of pMdRD29A-P1 in Fig. 7C.

Table S1 Primers used in this study

Primer Name	Primer Sequence
MdSND1-pRI	F: GTCGACATGTCAGATGATCAAATGAGT
	R: GGATCCTTACACCGACAAGTGGCAAAG
MdSND1-pGEX	F: GGATCCCCATGTCAGATGATCAAATGAGT
	R:GTCGACCACCGACAAGTGGCAAAGCGG
MYC-MdSND1-pRI	F: GTCGACATGGAACAAAAGTTGATTTCTGAA GAAGATCTTATGTCAGATGATCAAATGAGT
	R: GGATCCTTACACCGACAAGTGGCAAAG
RNAi-MdSND1	ZF:CTCGAGGCTATTCGGCCATTGACG
	ZR:GAATTCTGACGGTGATGACGACGA
	FF:TCTAGATGACGGTGATGACGACGA
	FR:AAGCTTGCTATTCGGCCATTGACG
**For EMSA probes**	
pMdAREB1A (SND1)	F: AATAAAAAAACATTTTTTATGGACGAAATTGCCCCGG
	R: CCGGGGCAATTTCGTCCATAAAAAATGTTTTTTTATT
pMdAREB1B (SND1)	F: AAAGAGTCAAGGCTTTCTTCCTAAGCAAGTAATAATA
	R: TATTATTACTTGCTTAGGAAGAAAGCCTTGACTCTTT
pMdDREB2A (SND1)	F: TCAGTGTTCAATTCTCCACCACACGGAATTGAACATG
	R: CATGTTCAATTCCGTGTGGTGGAGAATTGAACACTGA
pMdRD22 (SND1)	F: CACTTGCATTATCTTGAGGGACAAGGATTGTCTGCCC
	R: GGGCAGACAATCCTTGTCCCTCAAGATAATGCAAGTG
pMdRD29A (SND1)	F: ACTTCTATCAACTTTGACATCAAAGCAAGAGCTGCCC
	R: GGGCAGCTCTTGCTTTGATGTCAAAGTTGATAGAAGT
pMdAREB1A (SND1)	F: TCAGTGTTCAATTCTCCACCACACGGAATTGAACATG
	R: CATGTTCAATTCCGTGTGGTGGAGAATTGAACACTGA
pMdMYB46(SND1)	F: TATTATGATTACCTTGAGAATGAAGAAAATAGGTTTA
	R: TAAACCTATTTTCTTCATTCTCAAGGTAATCATAATA
pMdMYB83A(SND1)	F: TCGATTCATTTTTCTTCACTCACAAGGTAATCCTATAT
	R: ATATAGGATTACCTTGTGAGTGAAGAAAAATGAATCGA
pMdMYB83B(SND1)	F: ATGATTCATTTTTCTTCACTCACAAGGTAATCATGTAC
	R: GTACATGATTACCTTGTGAGTGAAGAAAAATGAATCAT
**For reporter genes**	
pMdAREB1A-P1 (SND1)	F: CTGCAG TAATTCAGATTGGATTGACCA
	R: GGATCCTGCTGGCCAATGAGATTGCGC
pMdAREB1B-P1 (SND1)	F:CTGCAG ATCCAATTAGGTAAAAAGGCA
	R:GGATCC TGGGTTGTGAACTTGTGATCA

pMdDREB2A-P2 (SND1)	F:CTGCAG CATCGTGCATCGTGTGGTCAG
	R:GGATCC CACGAACCATTTTTCACTACC
pMdRD22-P3 (SND1)	F:CTGCAGCAATTCAAGATGATCATTGAA
	R:GGATCCGTGTGGTCACGTATGTTAATA
pMdRD29A-P1 (SND1)	F:GTCGACAGTCTCCAGAGTCTCGGAGCC
	R:GGATCCCGTAATGCAGTTGATCATTGA
pMdMYB46（SND1）	F: GTCGACATTCATATGTTGGGCTTGGTT
	R: GGATCC GGGGTTGGTTGATGGGTCTGA
pMdMYB83A(SND1)	F: GTCGACATCCTCAATAAGTTTCATGCA
	R: GGATCCGCTTAAAATATGCATATCAAA
pMdMYB83B(SND1)	F: GTCGACAAAGGTGCAACACTTTGCCTA
	R: GGATCCGACCTCCGTGGCTCCATTCGC
pMdMYB46m (SND1)	F: TATTATGATTACCTTGAGAATGAATAAAATAGGTTTA
	R: TAAACCTATTTTATTCATTCTCAAGGTAATCATAATA
pMdMYB83Am (SND1)	F: TCGATTCATTTTTCTTCACTCACAATGTAATCCTATATC
	R: GATATAGGATTACATTGTGAGTGAAGAAAAATGAATCGA
pMdMYB83Bm (SND1)	F: TGATTCATTTTTCTTCACTCACAATGTAATCATGTAC
	R: GTACATGATTACATTGTGAGTGAAGAAAAATGAATCA
pMdAREB1A-P1m（SND1）	F: AATAAAAAAACATTTTTTATGGACTAAATTGCCCCGG
	R: CCGGGGCAATTTAGTCCATAAAAAATGTTTTTTTATT
pMdAREB1B-P1m（SND1）	F: AAAGAGTCAAGGCTTTCTTCCTAATCAAGTAATAATA
	R: TATTATTACTTGATTAGGAAGAAAGCCTTGACTCTTT
pMdDREB2A-P2m（SND1）	F: TCAGTGTTCAATTCTCCACCACACTGAATTGAACATG
	R: CATGTTCAATTCAGTGTGGTGGAGAATTGAACACTGA
pMdRD22-P3m (SND1)	F: CACTTGCATTATCTTGAGGGACAATGATTGTCTGCCC
	R: GGGCAGACAATCATTGTCCCTCAAGATAATGCAAGTG
pMdRD29A-P1m (SND1)	F: ACTTCTATCAACTTTGACATCAAATCAAGAGCTGCCC
	R: GGGCAGCTCTTGATTTGATGTCAAAGTTGATAGAAGT

For ChIP-qPCR

pMdAREB1A-P1	F: GTCCTCAAGGCCGACCGTTGT
	R: TGGTGGTGGCGACGGGATAAG
pMdAREB1B-P1	F: TAGTGACCTCACCATACACTA
	R: GCACTTTCGGGAGAAGGAGGT
pMdAREB1B-P2	F: CCACGTTTCGAGATCTGATCG
	R: GGAAAATTGAGGGGGTGACTG

pMdAREB1B-P3	F: CAGTAAGATTTAGAGAAACGG
	R: TCATCAGAAGCAATCAACTTA
pMdDREB2A-P1	F: TTTACCTGCATAATATGAACA
	R: ATGTTGATATGGTCATCGCAC
pMdDREB2A-P2	F: ATCTCTAAGATCGTCACAAAG
	R: AATGTGTAGGGCAACGTGGCA
pMdRD22-P1	F: GCGTTTTCAGCTCCCAATATA
	R: GACACGTAGTTGGAGACAGCA
pMdRD22-P2	F: CCAAGTAGGAAATGTGATGAA
	R: TCTAGAATTCTTCACTGTTTT
pMdRD22-P3	F: CAACCATCTTAATTAGGTGTG
	R: TGTTGACGTGGCTTAACCGTG
pMdRD29A-P1	F: GGTATACGAATACCACTAATT
	R: CGTATTATGTATCAGAACAAA
pMdMYB46	F:ATTCATATGTTGGGCTTGGTT
	R: TAGGGGTTGGTTGATGGGTCT
pMdMYB83A	F: TCTGCATATAAACTTCAATTT
	R: ATTATGTGAAGTGGAAGCAAA
pMdMYB83B	F: GCCTAGTGTACAAAGAGTACT
	R: TTATTATGTGAAGTGGAAGCA
**For qRT-PCR**	
MdDR22(DL)	F：TGATGGGGTGAATGTTAAAG
	R：GAACCCAGACAACATGATCA
MdSND1（DL）	F: ATCCTAATGATCAAGACA
	R: GCACTTCCTATATATATT
MdDR29A(DL)	F：CTGAAGAAGGTAAAGGAGAA
	R：CCTTCAAAATATCTCCTTGC
MdDR29B(DL)	F：CCAAATTACCATGCCTCAAC
	R：CCTTGGACTTGTACTCTCCC
MdAREB1A(DL)	F：CAGAGAATCAGCTGCCAGGT
	R：TCTCCATGTCCTGATTCTTC
MdAREB1B(DL)	F：TTAGAACTAGAGGCAGAAGT
	R：CTGTCAATGTTCGTCGTAAG
MdDREB2A(DL)	F：AAGAAAAGGAGGGGAGTAAT
	R：ATAGTTGTAACCTCCATCTC

MdDREB2B(DL)	F：CTCTAATTCTGTGTCATCTA
	R：AACATGTAATTCCTCTGATA
MdC4H(DL)	F: ACATGAACCTCCAGGATGCCA
	R: GAGATACCTGAAGTCGTTCCC
MdC3H(DL)	F: CTACTTCCATTTGGAGCAGGC
	R: CGGAGTAGGCACGACTTGGAC
MdCAD(DL)	F: ATGCAAGAAGCTGCTGACTCA
	R: GAAGCTCCCTGTGATTGTCTT
MdF5H(DL)	F: AGCCCTCTAGGTTTCTGAAAG
	R: GTCAAGCTCACTAGGTTTCAT
MdHCT(DL)	F: TGCTTTGGTGCGTATGGATGA
	R: CCAAAGTCGGCATCATGGATT
Md4CL(DL)	F: CCCTGATACGGGTGCTTCGCT
	R: CGGTCGACGATGAAGAGCTCG
MdCCR	F: TCACATTCTGGTCTATGAGAC
	R: CTTCTGGTTTGTGAACTTGTA
MdCOMT(DL)	F: GGTGAAGGTTGGGGGTTTGAT
	R: GATTCGACGGCAGAGAGTGAT
MdMYB46（DL）	F:CATCCTCACCTGCAATAAAAA
	R:GGTAGTCAAGAAAAGGAAATG
MdMYB83A（DL）	F: TTAACAACTACCACTTCCATA
	R: AGTATCCAGCATGAAACCCTC
MdMYB83B（DL）	F: CAGTTGGTAGCTGATAATATA
	R: ATCTTGCATGAAACCGTCGAA
MdActin	F: TGGTGAAGGCTGGATTTG
	R: CTGTGAGCAGAACTGGGTG
